# Large chondral fragment of the lateral femoral condyle treated with arthroscopic internal fixation in an elite young athlete: A case report

**DOI:** 10.1051/sicotj/2019041

**Published:** 2020-01-08

**Authors:** Mitchell W. Beckert, Robert G. Klitzman

**Affiliations:** 1 Department of Orthopaedics, Indiana University Indianapolis IN USA

## Abstract

Focal chondral lesions in the adolescent population create a particular challenge for orthopedic surgeons, and currently there exists no consensus on proper treatment. Numerous techniques for addressing focal chondral defects are employed in both pediatrics and adults, including fragment excision, debridement and fixation, bone marrow stimulation and microfracture techniques, cell-based options, as well as chondral and osteochondral grafts. Although historical evidence is mixed, recent reports of primary fixation of displaced cartilage fragments have shown favorable results. We present a case of reduction and fixation of a large displaced cartilage lesion in an elite young tennis player. Our results, in addition to other reports mentioned in this manuscript, highlight the importance of considering primary fixation of large chondral lesions when amenable to repair.

## Introduction

Focal chondral lesions in the adolescent population create a particular challenge for orthopedic surgeons due to their limited healing potential [1]. Cartilage restoration techniques have seen significant advances in the last decade; however, their role in pediatric patients is not well-defined due to the lack of high-quality, long-term data [2, 3]. While displaced osteochondral lesions amenable to fixation have shown good outcomes [4–6], the success of fixation when cartilage fragments are devoid of subchondral bone is rarely described and previously been questioned [7]. The evidence for reduction and internal fixation of large cartilage fragments is limited to case reports [8–15], and the purpose of this study is to contribute yet another case to the literature of successful primary fixation of a large chondral fragment in the knee, here in a highly active young patient.

Both the patient and parents provided written informed consent to the submission of this case report.

## Case report

An 11-year-old elite level male tennis player presented for evaluation of acute right knee pain after jumping and landing on his feet during a tennis tournament. The patient was accompanied to clinic by his father, who reported immediate pain, swelling, and locking of his knee after landing. He was unable to continue participation or bear weight on the affected leg. The patient denied any history consistent with patellar subluxation or dislocation. At the time the patient was the number two ranked tennis player in the United States for his age, and had traveled across the country to compete against players from all over the world. He was otherwise healthy and had no significant past medical, surgical, or relevant family history. He was a very active competitive athlete in the seventh grade.

The patient was first evaluated at an outside facility, where MRI demonstrated a large chondral defect sitting in the femoral notch. He subsequently was referred to a pediatric orthopedist who placed him in a knee immobilizer, recommended not to bear weight on the affected side, and referred the patient to see us in the orthopedic sports medicine clinic. The patient was 5 feet 1 inch tall, and weighed 44.6 kg at the time of examination. On physical exam, his right knee demonstrated a mild effusion. Range of motion testing was deferred to avoid further cartilage damage; however, the patient was comfortably resting at approximately 90° of flexion and reported being able to bear weight on the right leg. He was neurovascularly intact. Examination of the contralateral knee was within normal limits, with range of motion negative 3–135°.

**Figure 1:**
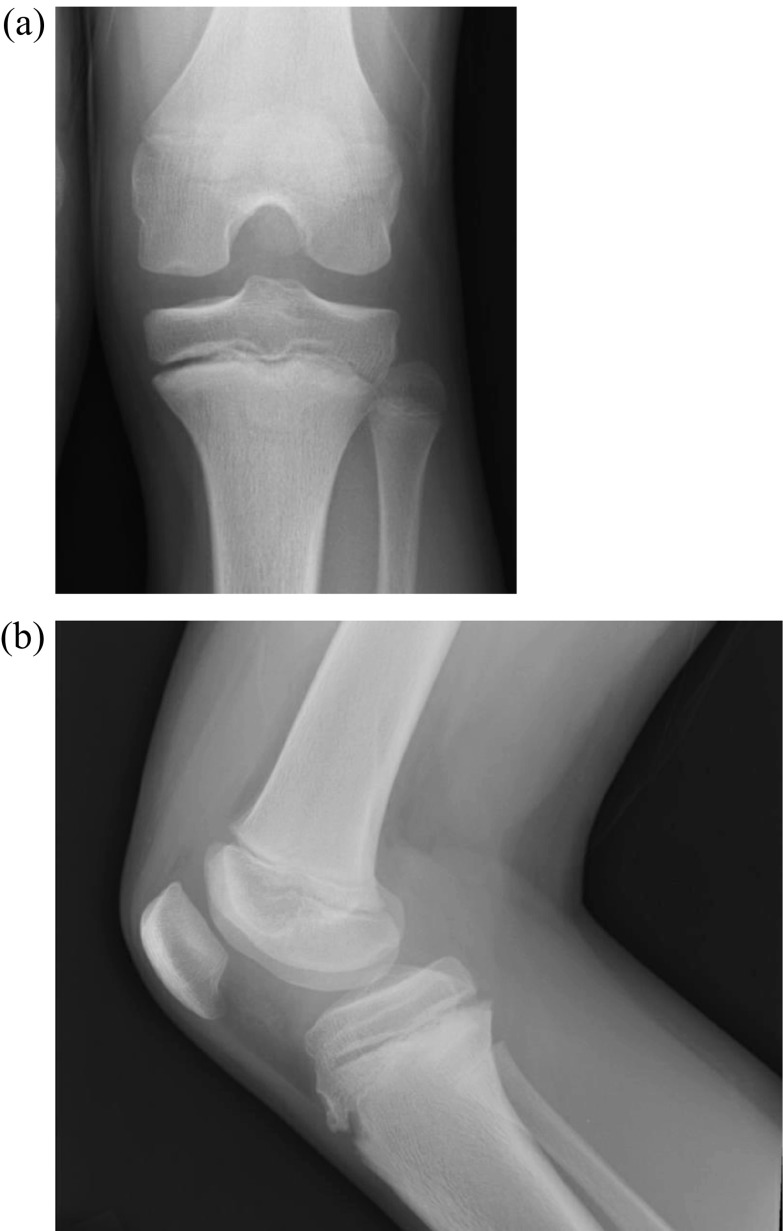
Preoperative AP and lateral radiographs unremarkable for bony pathology (a and b).

**Figure 2:**
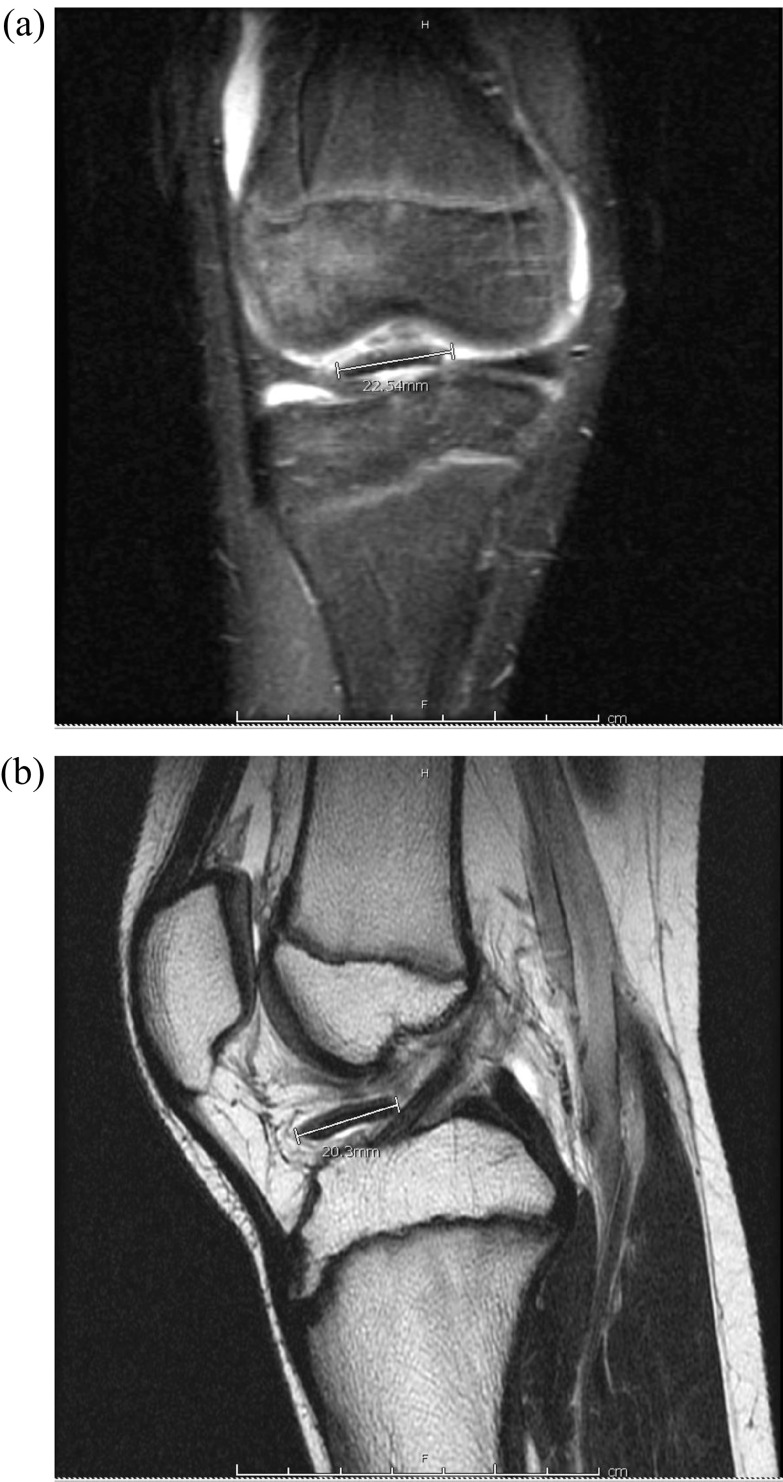
T2- and T1-weighted MRI demonstrating large chondral fragment within the intercondylar notch (a and b).

**Figure 3:**
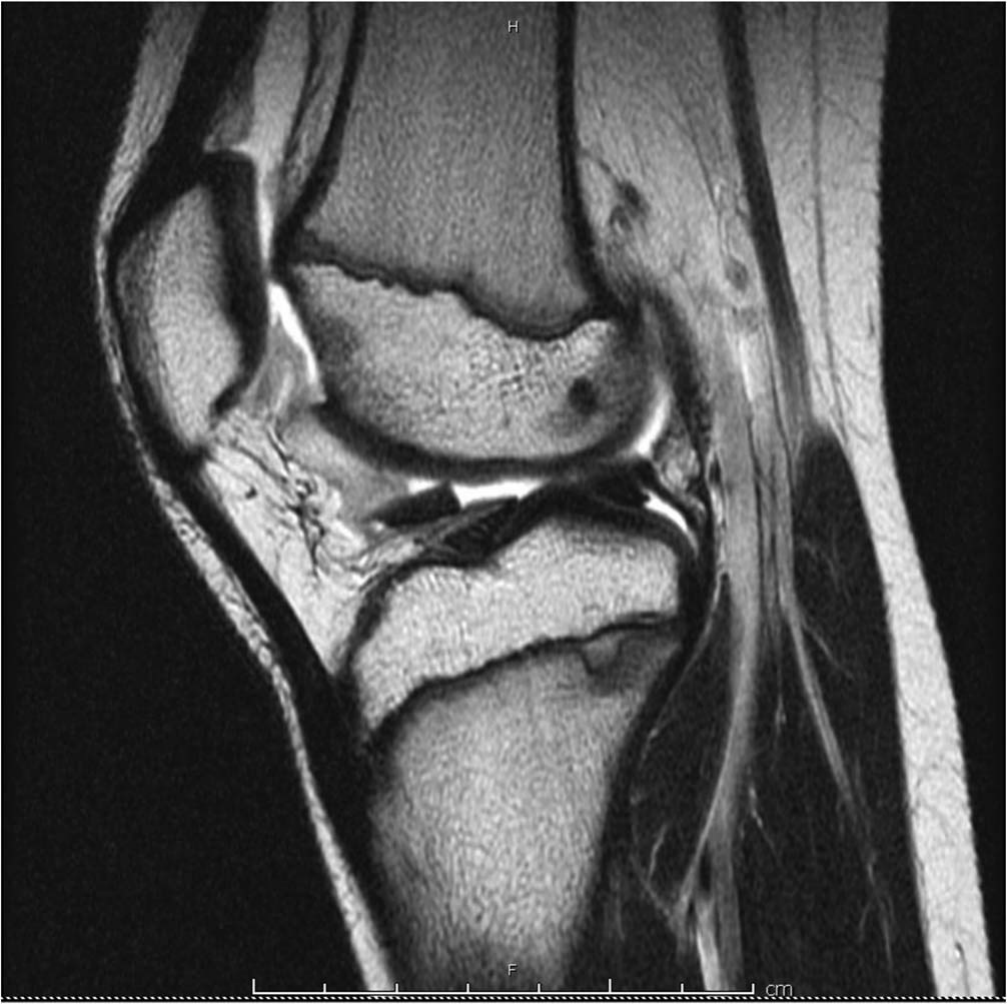
T1-weighted MRI showing full-thickness cartilage defect at the lateral femoral condyle.

Radiographs obtained were normal without fracture or dislocation (Figures 1a and 1b). MRI demonstrated a 22 × 20-mm chondral defect originating from the lateral femoral condyle that was now sitting in the femoral notch (Figures 2a and 2b). Likewise, the lateral femoral condyle showed a full thickness defect with no evidence of injury to the subchondral bone (Figure 3). There was no evidence of injury to the patellar cartilage or medial soft tissue restraints to suggest acute patellar dislocation. A lengthy discussion was had with the patient and father regarding the significance of this injury and need for surgical fixation to maximize both short and long term functional status of his knee. Consent for surgery was obtained and scheduled for 6 days after initial consultation. We stressed the importance of maintaining a non-weight bearing status to his right lower extremity in the interim to minimize risk of further damage to this loose cartilage fragment.

**Figure 4:**
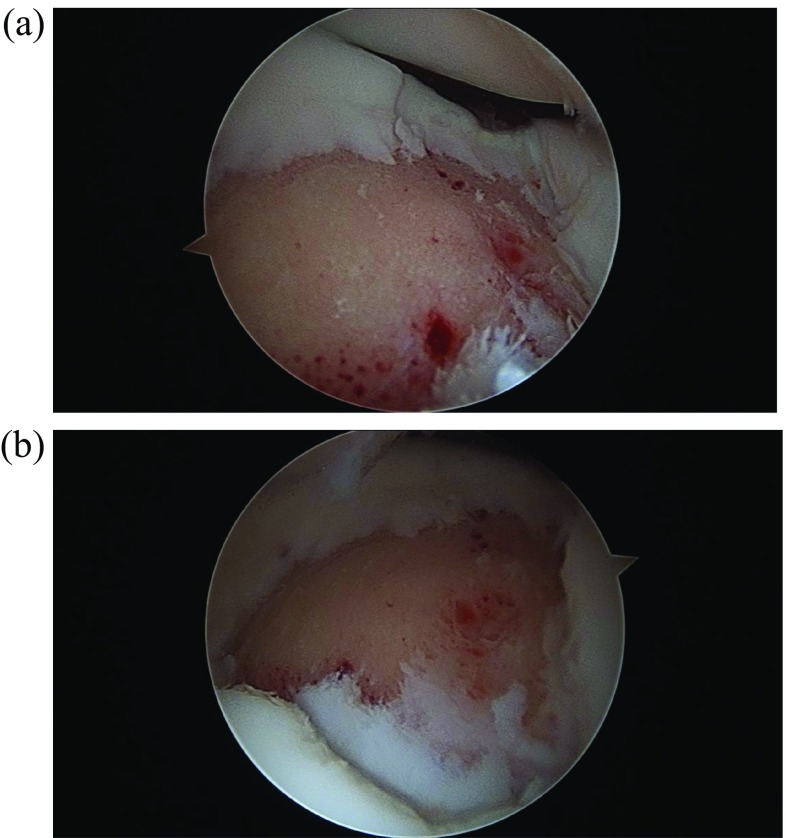
Intraoperative arthroscopic images showing large full-thickness cartilage defect at the lateral femoral condyle without disruption of the underlying subchondral bone (a and b).

**Figure 5:**
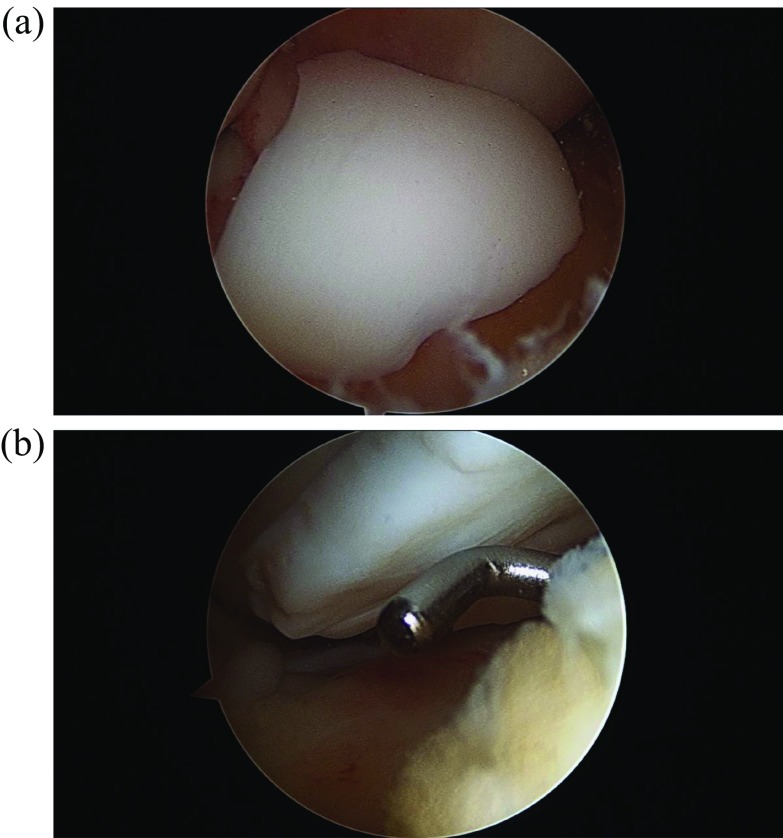
Intraoperative arthroscopic images showing the large loose chondral fragment. This was found in one large piece within the intercondylar notch, without underlying bony attachment (a and b).

**Figure 6:**
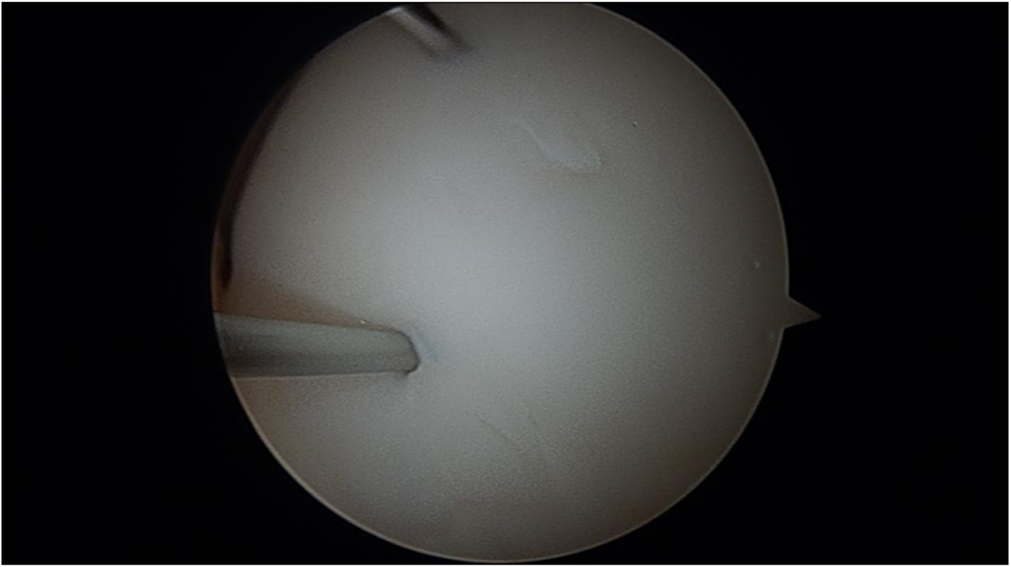
Provisional fixation of the chondral fragment using k-wires.

**Figure 7:**
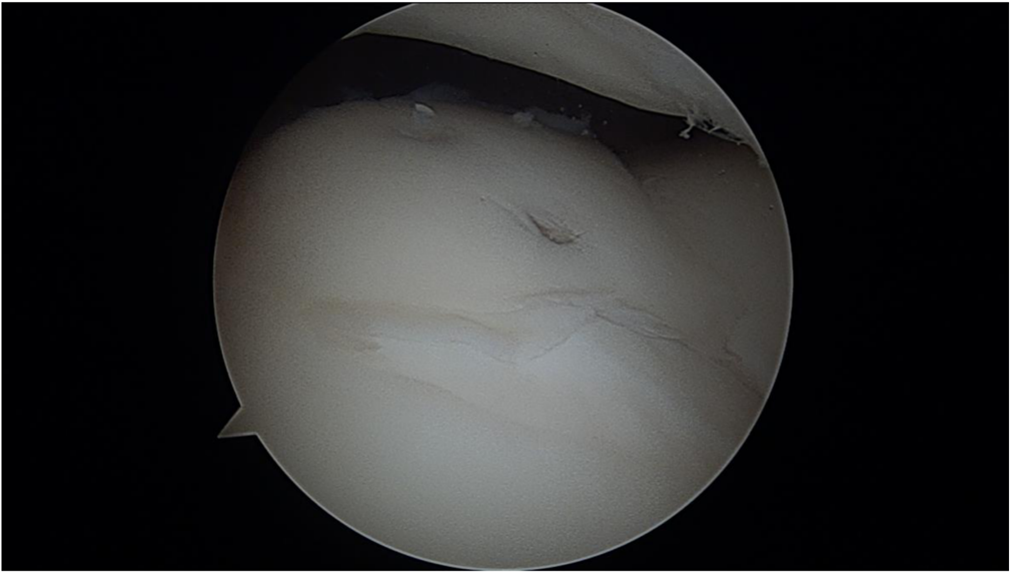
Definitive fixation of the chondral fragment using three bioabsorbable pins in a triangular fashion.

The patient was taken to surgery. An exam under anesthesia was then conducted. He was stable to varus and valgus stress and could achieve full extension. Flexion was not tested to protect his loose fragment within the knee. A diagnostic arthroscopy then took place. He was found to have no cartilage damage to the undersurface of the patella. No loose bodies were noted in the suprapatellar pouch, medial or lateral gutters. He had full-thickness cartilage loss of almost the entire upper lateral femoral condyle (Figures 4a and 4b). It did have full shoulders around the edges, where the cartilage was lost. It was greater than 2 × 2 cm. No cartilage damage was found to the rest of the lateral femoral condyle or the lateral tibial plateau. No cartilage damage to the medial femoral condyle or medial tibial plateau was appreciated. The medial and lateral menisci were intact without tears. Moving to the notch, the ACL and PCL were intact. The entire cartilage fragment was in one piece and sitting in the notch (Figures 5a and 5b). Next, curettes were used to scrape away the calcified layer over the exposed bone from the donor site of the cartilage on the lateral femoral condyle. The cartilage piece was then reduced and held in place with k-wires (Figure 6). Fixation was achieved with three absorbable Biotrak Helical Nails^©^ (Acumed, LLC, Hillsboro, Oregon, USA) in a triangular fashion to the underlying subchondral bone (Figure 7). The piece was then probed and found to be stable. Temporary fixation pins were removed, and the knee was ranged through a full arc of motion. The patella was observed to track well over the fixed piece. He was placed in a knee brace locked in extension immediately postoperatively.

The patient was discharged from the hospital the same day with crutches and strict non-weight bearing status, using the brace during ambulation. The patient returned to clinic 2 weeks postoperatively where sutures were removed, and a continuous passive motion device was given to help improve his knee range of motion. He was given no restrictions to knee range of motion. At 6 weeks postoperatively the patient was allowed to begin bearing weight on his right leg with the brace locked in extension using crutches. Formal physical therapy was initiated at this time. At 8 weeks postoperatively the patient was allowed to walk with his brace unlocked with the assistance of crutches. At 10 weeks postoperatively he began walking in his brace without crutches. At 3 months postoperatively the patient reported beginning to hit tennis balls with his feet flat on the ground without pivoting on his right leg. At 4 months postoperatively the patient had weaned out of his brace and continued to hit tennis balls from a stationary position. He was allowed to slowly increase his activities at this time starting with a light jog and subsequently cleared to return to full activities at 6 months postoperatively.

**Figure 8:**
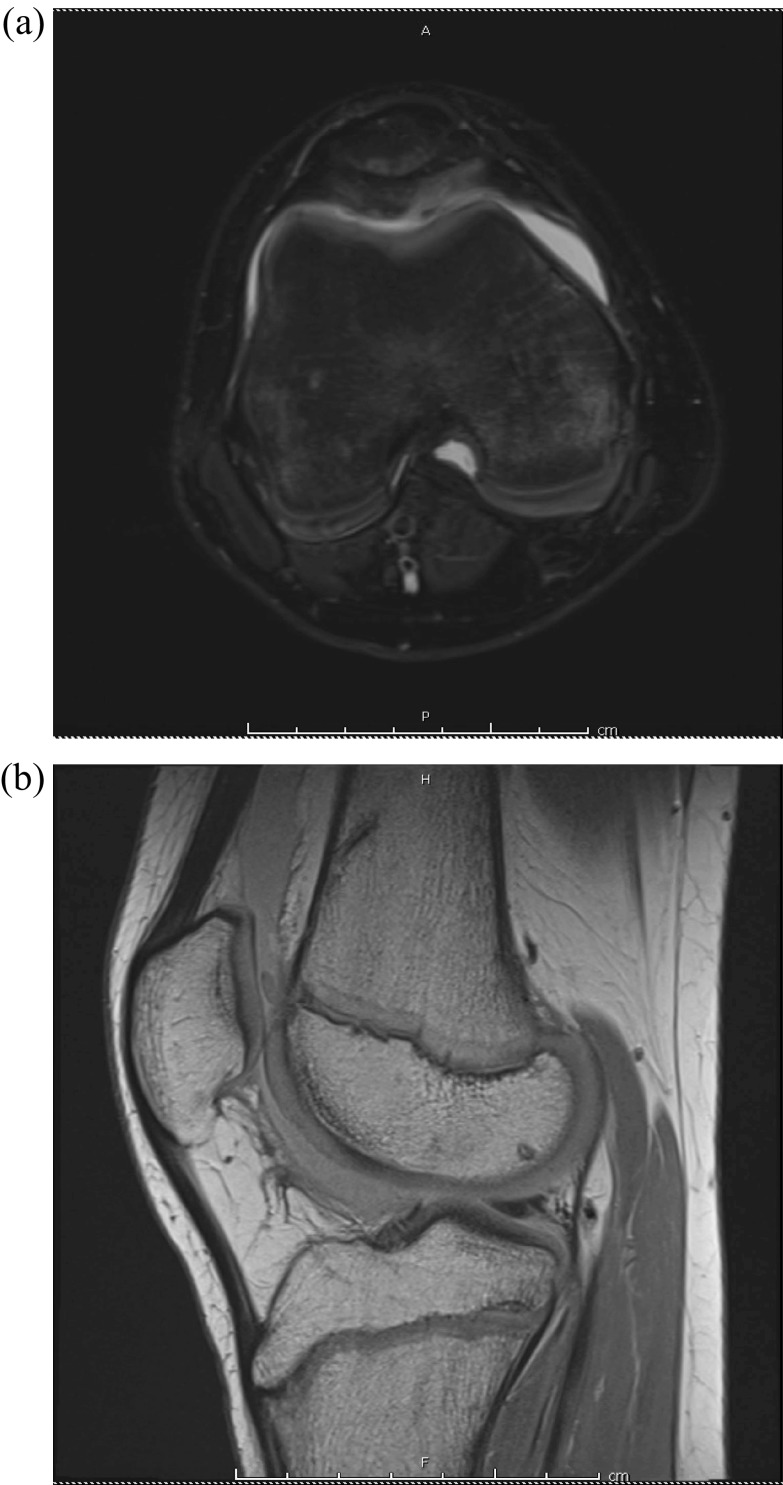
Axial T2- and sagittal proton density-weighted MRI showing incorporation of the previously fixed chondral fragment with near anatomic articular surface (a and b).

**Figure 9:**
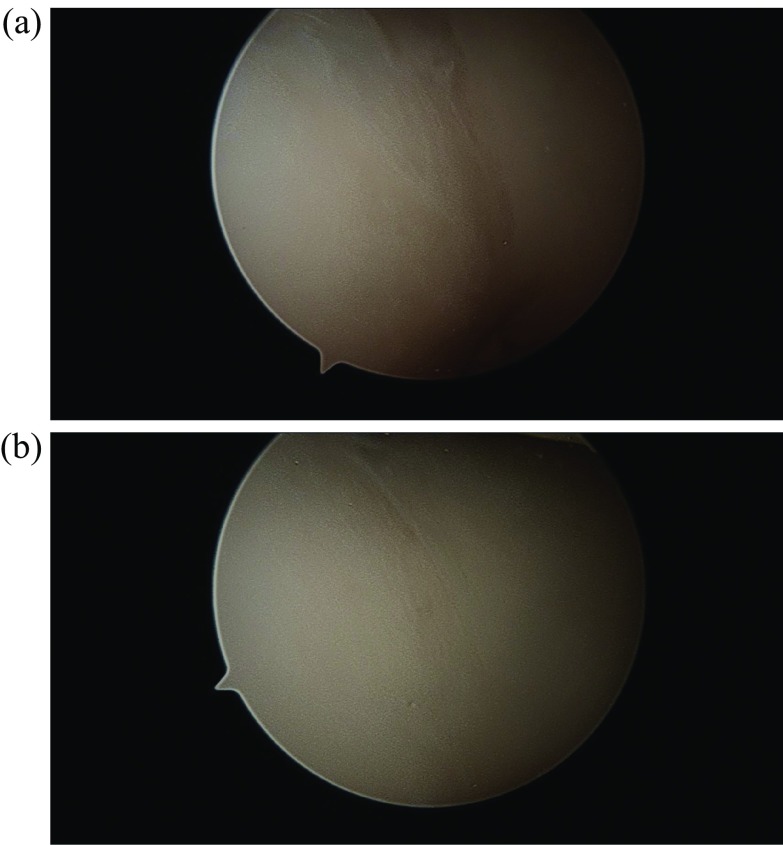
Second look arthroscopy showing excellent incorporation of the previously fixed chondral fragment with minimal articular cartilage irregularities (a and b).

By 7 months postoperatively the patient had played in two tennis tournaments, winning the second tournament. The patient was seen again 10 months out from his surgery and had recently returned from playing a clay court tournament in France. His right knee range of motion was −3° to 140° and symmetric to the contralateral side. He had full quadriceps and hamstrings strength and complained of no pain or swelling. The patient returned to competitive sports; however, several months later he began complaining of some posterior right knee pain as well as intermittent swelling. MRI was obtained, now a year and a half out from the initial surgery, which showed a posterior medial meniscus tear with maintained reduction and apparent incorporation of the previously fixed chondral fragment (Figures 8a and 8b). He subsequently underwent repeat right knee arthroscopy and medial meniscus repair. At the time of arthroscopy (1 year, 9 months from the index procedure), the site of chondral fixation was found to have excellent incorporation with a nearly anatomic articular surface (Figures 9a and 9b). He rehabbed appropriately and recovered well from this injury. At final follow-up (2 years, 7 months) the patient had no pain, ranked in the top five nationally for his age, and continued to compete at an international level.

## Discussion

Osteochondral injuries in the pediatric population are well-documented and most commonly caused by trauma, often following acute patellar dislocation, or a result of osteochondritis dissecans (OCD) [16–22]. Although osteochondral lesions usually have some degree of subchondral bone attachment, a cartilage fragment can occur in isolation. Previous biomechanical studies have shown decreased resistance to shear stress at the osteochondral junction in adolescents compared to adults, making adolescents more susceptible to this type of injury [23, 24]. When a displaced fragment is purely cartilaginous, it creates a difficult dilemma for the treating physician with no clear consensus option. Various treatment options for chondral defects include fragment excision, debridement and fixation, bone marrow stimulation and microfracture techniques, cell-based options, as well as chondral and osteochondral grafts [2]. Goals of treatment are focused on restoring articular congruity of the joint surface and preventing future osteoarthritis [2, 21, 22].

Fixation has historically been indicated for the classic osteochondral defect with a true osseous component, while excision with or without restorative procedures reserved for the cartilage-only fragment [25, 26]. Several authors have considered these cartilage fragments “unsalvageable” and routinely excise during surgery and are discarded [8, 25, 27–33]. Maletius and Lundberg reported poor healing potential in two cases of chondral fragment fixation, subsequently questioning the efficacy of this technique [7]. Recent reports, however, have shown successful fixation of purely cartilaginous lesions [8–15]. All cases were performed with open arthrotomies, except one case of a 22-year-old patient who underwent fixation of a 1.5 cm^2^ cartilage fragment with arthroscopy [15]. Previously described methods of fixation of these cartilage fragments include bone pegs, chondral darts, bioabsorbable nails, and headless screws, of which can be augmented by fibrin glue or suture [8–15].

Here we contribute another case of a large displaced osteochondral lesion completely devoid of bone that was successfully treated by native fragment reduction and fixation. Additionally, to our knowledge, this is the only case of successful fixation of a chondral-only fragment of this size by arthroscopic means.

## Conclusion

Large displaced cartilage fragments in the pediatric knee without attachment of subchondral bone create a difficult problem for the orthopedic surgeon and patient. This case and other case reports previously mentioned highlight the importance of considering primary fixation as a treatment option for adolescents with this specific injury pattern. For young patients, the senior author (RGK) recommends arthroscopic reduction and fixation of displaced chondral and osteochondral lesions that are amenable to fixation to restore the native articular congruity.

## Conflicts of interest

The authors have no conflicts of interest to report.
